# Biofunctional coatings and drug-coated stents for restenosis therapy

**DOI:** 10.1016/j.mtbio.2024.101259

**Published:** 2024-09-19

**Authors:** Yanghui Wen, Yihuan Li, Rui Yang, Yunjie Chen, Yan Shen, Yi Liu, Xiaomei Liu, Botao Zhang, Hua Li

**Affiliations:** a Departments of General Surgery, Ningbo No.2 Hospital, Ningbo, 315010, China; b Zhejiang Engineering Research Center for Biomedical Materials, Laboratory of Advanced Theranostic Materials and Technology, Ningbo Institute of Materials Technology and Engineering, Chinese Academy of Sciences, Ningbo, 315201, China; c Zhejiang-Japan Joint Laboratory for Antibacterial and Antifouling Technology, Ningbo Institute of Materials Technology and Engineering, Chinese Academy of Sciences, Ningbo, 315201, China

**Keywords:** Stent, Drug-coated stents, Surface modification, Coating, Interventional therapy

## Abstract

Palliative therapy utilizing interventional stents, such as vascular stents, biliary stents, esophageal stents, and other stents, has been a prevalent clinical strategy for treating duct narrowing and partial blockage. However, stent restenosis after implantation usually significantly compromises therapeutic efficacy and patient safety. Clinically, vascular stent restenosis is primarily attributed to endothelial hyperplasia and coagulation, while the risk of biliary stent occlusion is heightened by bacterial adhesion and bile sludge accumulation. Similarly, granulation tissue hyperplasia leads to tracheal stent restenosis. To address these issues, surface modifications of stents are extensively adopted as effective strategies to reduce the probability of restenosis and extend their functional lifespan. Applying coatings is one of the technical routes involving a complex selection of materials, drug loading capacities, release rates, and other factors. This paper provides an extensive overview of state of the art drug-coated stents, addressing both challenges and future prospects in this domain. We aim to contribute positively to the ongoing development and potential clinical applications of drug-coated stents in interventional therapy.

## Graphical abstract

**Figure:**
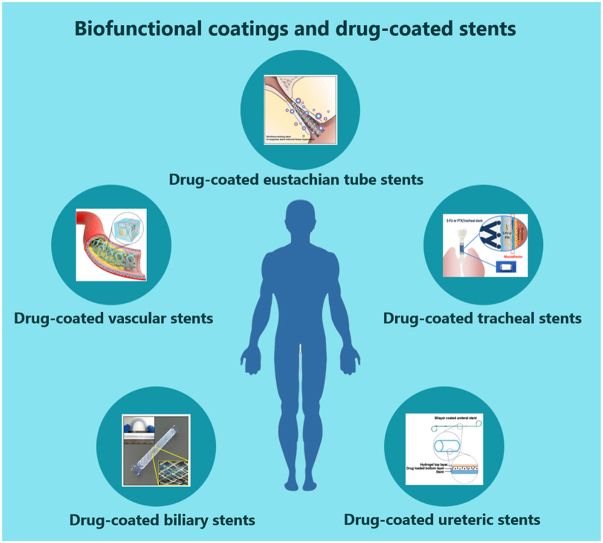

## 1 Introduction

Stents used in clinical practice usually pertain to cardiovascular stents, tracheal stents, intestinal stents, esophageal stents, urethral stents, pancreatic stents, biliary stents, and neural scaffolds [[1], [2], [3], [4]]. However, traditional stents are prone to various problems in clinical applications, such as restenosis of stents [[5], [6], [7], [8], [9]], rapid degradation rate or fracture of stents [10,11], and function deficiency of stents [12]. Continuous innovation in surface functionalization and coating for stents [[13], [14], [15], [16], [17]] have emerged as inevitable outcomes of advancements in materials science and biotechnology, tackling these challenges effectively. Progress in the design and delivery systems of novel drug-coated stents has resulted in extraordinary benefits, establishing them as a minimally invasive therapeutic approach.

Therefore, comprehensive understanding of stent occlusion is essentially required for the designing and manufacturing of ideal interventional prosthetic devices. Within milliseconds of the artificial scaffold coming into contact with biological fluids such as blood, bile and tracheal mucus, the scaffold surface usually adsorbs a layer of key proteins, mediating the biological reaction to the biological material. The presence and function of macromolecules and subsequent cell/tissue interactions are crucial to the long-term properties and functionalities of implants [18]. Blood coagulation, biliary stent occlusion, and mucus plugging are generally undesired interactions in stent interventional therapy. Typically, several plasma proteins, such as fibronectin, fibrinogen, and vitronectin, and von Willebrand factor mediate platelet adhesion and induce thrombosis [19]. These adsorbed proteins on the scaffold can further induce platelet adhesion, activation, aggregation and thrombosis by binding to the platelets through receptors on the cell membrane [19]. Surface modification techniques significantly suppress the formation of blood clots or thrombi, a critical issue for vascular stents in direct contact with blood [20,21].

Until now, commercially available self-expandable metal stents (SEMS) and plastic stents are the ideal biliary stents used to prevent biliary obstruction. However, stent occlusion remains a common problem for both types of stents. Early stent occlusions within 30 days are mostly caused by mispositioning, debris, blood clots or mucus produced by mucus-secreting tumor [22,23]. The mechanism of late stent occlusion is associated with bacterial biofilm and biliary sludge which mainly consists of calcium bilirubinate and calcium palmitate crystals formed by bacterial enzymes [24,25].

Furthermore, problems may arise with the use of ureteral stents in the urinary tract due to biofilm formation and the accumulation of mineral salts on the stent surface [17,26,27]. Adsorption of complex biological molecules (proteins, lipids, polysaccharides) usually occurs along the surface of the stent within minutes after implantation and induces bacterial recruitment, urinary salt deposition and stone formation [26,28]. Prolonged encrustation occurs within days of stent placement, leading to obstruction of the stent lumen and renal unit, stent fracture, urosepsis, and even loss of renal function.

In the abovementioned cases, a mixed strategy with drug-coated stents has been developed to control the surface behaviors of artificial medical implants [[29], [30], [31], [32], [33], [34], [35]]. The drug-coated stent features a modified surface equipped with a drug-releasing structure to regulate the interface responses. A typical drug-coated stents consists of a metal stent, drug-loading matrix, drug, and is mostly coated with specific polymers or other materials on the bare metal scaffold surface, conjugated with a drug [1,6,16,29,33], antibody [36], or gene [37]. The drug-containing coating serves as an intermediate functional layer between the scaffold and surrounding tissue. After implantation, the drug is released in the diseased lumen to inhibit the undesired conditioning film adsorption, body fluid adhesion, cellular proliferation, blood platelet attraction and activation, bacterial attachment/biofilm formation, crystal deposition and immunological rejection. Therefore, drug-coated stents show promising clinical efficacy for long-term applications as compared to traditional bare stents.

In a typical drug-coated vascular scaffold, the coating matrix is usually made with permanent polymers such as poly(n-butyl methacrylate) (PBMA) [39], phosphorylcholine (PC) [38], and biodegradable polymers such as poly(lactic acid) (PLA) [40,41], poly(ε-caprolactone) (PCL) [40], poly (lactic-co-glycolic acid) (PLGA) [40,42], and poly[α-(4-aminobutyl)-L-glycolic acid] (PAGA) [43]. Based on their mechanism of action, immunosuppressive drugs like rapamycin [44,45], anti-proliferative drugs like paclitaxel [46,47], antithrombotic drugs like heparin [48], and anti-inflammatory drugs like dexamethasone [49] have been loaded on the stent surfaces.

Drug-coated stents exhibit outstanding dredging efficiency in eliminating duct narrowing/partial blockage and markedly reduce the restenosis rate by regulating the physicochemical properties of the stent surface and controlling the delivery modes and release rate of the loaded drugs [50]. Drug-coated stents can not only inhibit the proliferation of vascular smooth muscle cells, improve the antithrombotic property of stents, inhibit bacterial infection and deposition of bile sludge, and ensure the effectiveness of stents, but also increase the corrosion resistance of the stents, protect against the leaching of metal ions, and prevent the occurrence of complicated immune, blood, and tissue reactions between the bare-metal stent and the host [29,32,[44], [45], [46], [47], [48], [49], [50]].

This paper provides a comprehensive overview of materials and coating technologies of drug-coated stents: cardiovascular stents, digestive tract stents, respiratory stents, reproductive system stents, otolaryngology stents, neural scaffold and lymphatic stents (Table 1). Moreover, we critically review the occlusion mechanisms and complications of stent placement. Additionally, we elucidate the functional coating design, surface modification processes, and biological performances of newly developed drug-coated stents. We conclude by discussing challenges and opportunities for the future of drug-coated stents in implantation or intervention therapies.

**Table 1:** Surface modified stents for restenosis therapy.

**Category**	**Type**	**Materials/Microstructure**	**Method of fabrication**	**Drugs**	**Commercially available DES**
Cardiovascular stent	Permanent polymer coating	PSIBS; PEVA; PBMA; PVDF; PVDF-HFP; PHFP; BioLinks	Microdrop spray; Electrophoretic deposition; Self-assembly method	Sirolimus Paclitaxel Everolimus Zotrolimus	Cypher; Taxus; Xience; Endeavour; Resolute
	Polymer free	Porous hydroxyapatite coating; Micropores; Grooves; Texture surface; holes	Laser Sculpted Drilled Microblasting	Sirolimus Probucol Paclitaxel	Yukon Chrome; ISAR; Janus Flex; Amazonia pax; Conor Stent; CoStar; Nevo; Medtronic's drug filled stent; VESTAsync
	Biodegradable coating	PLLA; PDLLA; PDLLA-PCL; PLGA; PLA-PCL; Magnesium Hydroxide; Chitosan; Heparin	Microdrop spray; Electrostatic spinning	Sirolimus Novolimus Everolimus Biolimus Paclitaxel Myolimus Salicylate	Synergy; Orsiro; Absorb; Absorb-GT1; MeRes; Magmaris; Biomatrix alpha; Ultimaster; Yukon Choice PC; DESyne BD; Combo; Mistent; BVS V.1.0 and BVS V.1.1; DESolve; ReZolve and REVA Gen; IDEAL; ART 18Z; Xinsorb BRS
Digestive tract stents	Fully covered film	Permalume coating; Partially silicone coating	Covering	N/A	Wallflex; Niti-S; ENDO-FLEX Self-Expanding Nitinol Stents; Ultraflex
	Permanent polymer coating	PU	Spray	Gemcitabine; Paclitaxel;	N/A
	Biodegradable coating	PLLA; PLGA PCL; PEG;	Spray; Electrostatic spinning	Gemcitabine; Paclitaxel; Sorafenib; Vorinostat	N/A
Respiratory stents	Fully covered film	Silicone coating	Covering	N/A	Ultraflex; AERO; Bonastent
Reproductive system stents	Biodegradable lubricious coating	Povidone-iodine-coating	Dip coating; Chemical modification	N/A	INLAY
	Antibacterial coating	Biocide coating; Surface texture	Dip coating; Spray	Ag, Furosulacillin; Rifamomycin; Sparfloxacin; Minocycline; Gentamicin	Bardex I.C.; Lubri Sil I.C.; Dover; Radiance Sharklet;
	Anti-tumor coating	PCL	Immersion	Epirubicin; Paclitaxel; Gemcitabine; Doxorubicin	N/A
Otolaryngology stents	Biodegradable anti-hyperplasia coating	PLGA	Ultrasonic spray	Sirolimus	N/A

## 2 Cardiovascular stent

The leading cause of death and disability globally, cardiovascular disease (CVD) accounts for nearly 30 percent of all deaths worldwide [51]. Currently, coronary stenting is the most commonly used effective method to treat cardiovascular stenosis, which mainly involves dilating the catheter with a balloon and then implanting a stent into the stenotic area of the blood vessel to unclog the arterial blood vessels [5]. After permanent stent implantation in stenotic vessels, the artificial stent acts as a foreign body promoting platelet activity. Patients are usually required to take antiplatelet drugs for an extended period to prevent thrombosis. However, there are some potential risks, such as late stent thrombosis [14,52], anaphylaxis [53], in-stent restenosis [54], and others. Generally, within 6 months of stent implantation, severe restenosis may act as a trigger for subacute vessel occlusion. The previous stent must be removed and re-implanted for reconstruction.

**Fig. 1:**
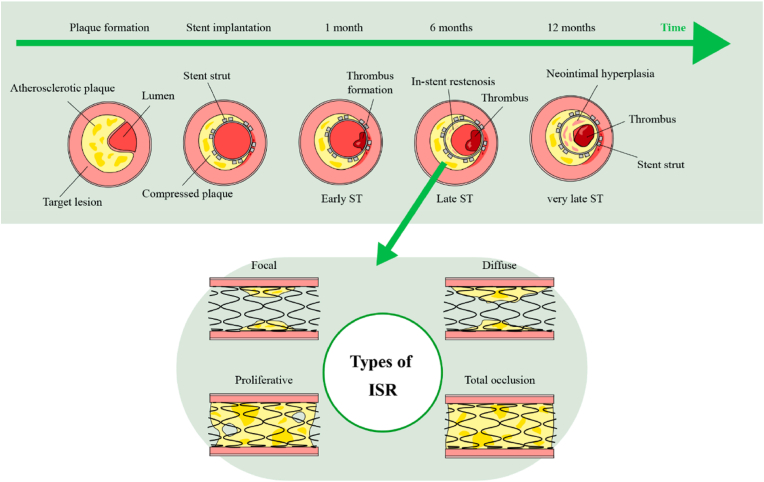
The development process of in-stent restenosis and stent thrombosis after stent implantation [56].

The main reasons for restenosis after stent implantation lie in two aspects: (1) Stent expansion may injure endothelial cells, thereby triggering the proliferation and fibrosis of vascular smooth muscle cells as well as endothelial hyperplasia. If the endothelium grows excessively, restenosis will inevitably occur [54]. (2) Blood serum proteins are automatically adsorbed onto the surface of the scaffold. If the adsorption layer exceeds a specific thickness, further platelet and erythrocyte adhesion is induced. The rupture of the adhered red blood cells can release coagulation factors such as adenosine diphosphate and erythrocyte, ultimately leading to the coagulation reaction [18,19,55]. In addition, proteins adsorbed on the stent surface activate exogenous and endogenous coagulation systems in the blood, triggering chemical reactions that lead to thrombus formation on the stent. Another adverse effect is neointimal hyperplasia caused by the excessive proliferation of the smooth muscle cells, which is an inflammatory response to the stent as a foreign material. This implies that when the implanted stent malapposition could cause early stent thrombosis (<1 month post-implantation), late stent thrombosis (1–12 months post-implantation), and very late stent thrombosis (>12 months post-implantation) (Fig. 1) [56]. Therefore, rapid re-endothelialization, anti-thrombosis, and anti-inflammation are the critical issues for the long-term effectiveness of vascular stents.

**Fig. 2:**
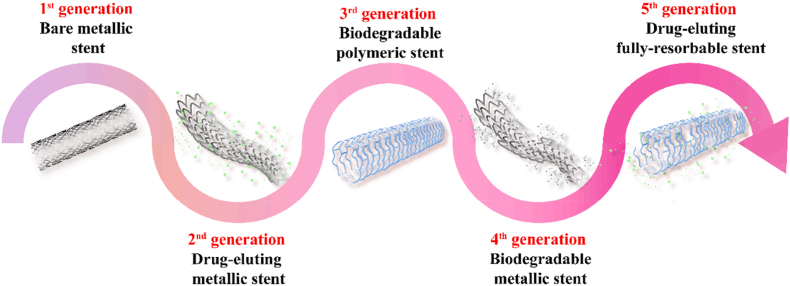
The evolution of first-to fifth-generation vascular stents [56].

The stent has steadily evolved from the first-generation bare metal stents to second-generation drug eluting stents, third-generation biodegradable polymetric stents, fourth-generation biodegradable metallic stents and fifth-generation drug-eluting fully-resorbable stents (Fig. 2) [56]. Drug eluting stents maintained its superiority over bare metal stents in restenosis rate and late lumen loss, which initially utilized with thick drug coatings on stainless steel stents changed to thinner struts and thin coatings, contained drugs changed from Paclitaxel and Sirolimus to Zotrimoxazole and Everolimus [57], and used biodegradable polymer and eventually moved towards polymer-free drug coatings [58]. Consequently, the development of novel materials and drug‐loading coated surfaces, as well as surface modification approaches of regenerative stents are critical strategies to solve the problems of restenosis and thrombosis in implanted stents *in vivo*.

### 2.1 Drug-coated vascular stents

Surface engineering strategies for improving anti-thrombosis, anti-inflammation, and selective endothelial cells simultaneously are essential for addressing the clinical complications associated with restenosis and late stent thrombosis and creating a microenvironment conducive to re-endothelialization [13,36,50]. The polymer stents with biodegradable polymetric coatings or durable polymer coatings for drug eluting and polymer-free stents are extensively utilized in coronary vascular intervention. The biodegradable drug carrier, such as Poly Lactic-co-Glycolic Acid (PLGA), poly(lactic acid) (PLA) and poly (lactic acid) (PLLA), were developed to suppress durable polymer induced inflammatory reactions and restrain late thrombosis and very late thrombosis. Drug-eluting stents loaded with anti-proliferative drugs like Paclitaxel, Sirolimus, Simvastatin, Zotrolimus and Everolimus were developed to inhibit neointimal hyperplasia [41,46,47]. Recent advances encompass a paradigm shift toward surface modified coatings to improve stent efficacy, which include coatings for controlled drug delivery, nanofibers (NFs)-coated stents and nanoparticles (NPs)-eluting stents, polymer-free techniques and drug-free nanotopographical approaches.

**Fig. 3:**
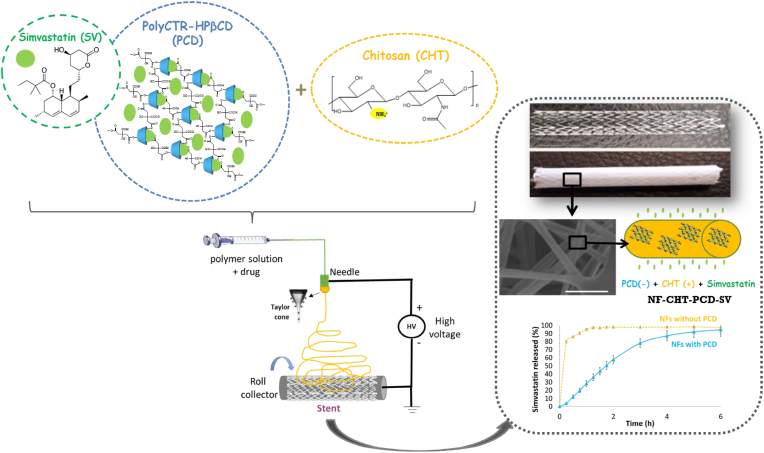
Schematic diagram of a simvastatin (SV) loaded drug-eluting stent covered with biodegradable nanofibers (NFs) based coatings [59].

The biodegradable NFs based coatings on DES stents possess porous structure and offer high surface-area-to-volume ratio for incorporating drugs or biologics, which can mimic the structure of natural extracellular matrix and provide desirable microenvironment for induced material-cell interaction. Chitosan (CHT)-based coating is a natural polysaccharide polymer that has outstanding biodegradability and antibacterial properties. The statins can offer protection to endothelial cells through their anti-inflammatory and antioxidant effects, as well as their action on the nitric oxide synthase system. Lille et al. developed a covered stent coated with NFs based on CHT and β-cyclodextrin polymer (PCD) with a high surface-area-to-volume ratio and a porous structure (Fig. 3) [59]. The PCD and simvastatin (SV) were dissolved in 90 % aqueous acetic acid solution. Then, an electrostatic spinning solution was prepared by adding CHT and polyethylene oxide (PEO). The SV-loaded CHT/PCD-based NFs-coated stents were then prepared using the electrostatic spinning technique. Cyclodextrin polymers with hydrophobic cavities can create reversible inclusion mixtures with lipid-friendly drugs, thus enhancing drug solubility and bioavailability. The nitinol stent covered with CHT/PCD/SV NFs coating reduces the multiplication of smooth muscle cells and improves endothelial cell multiplication.

**Fig. 4:**
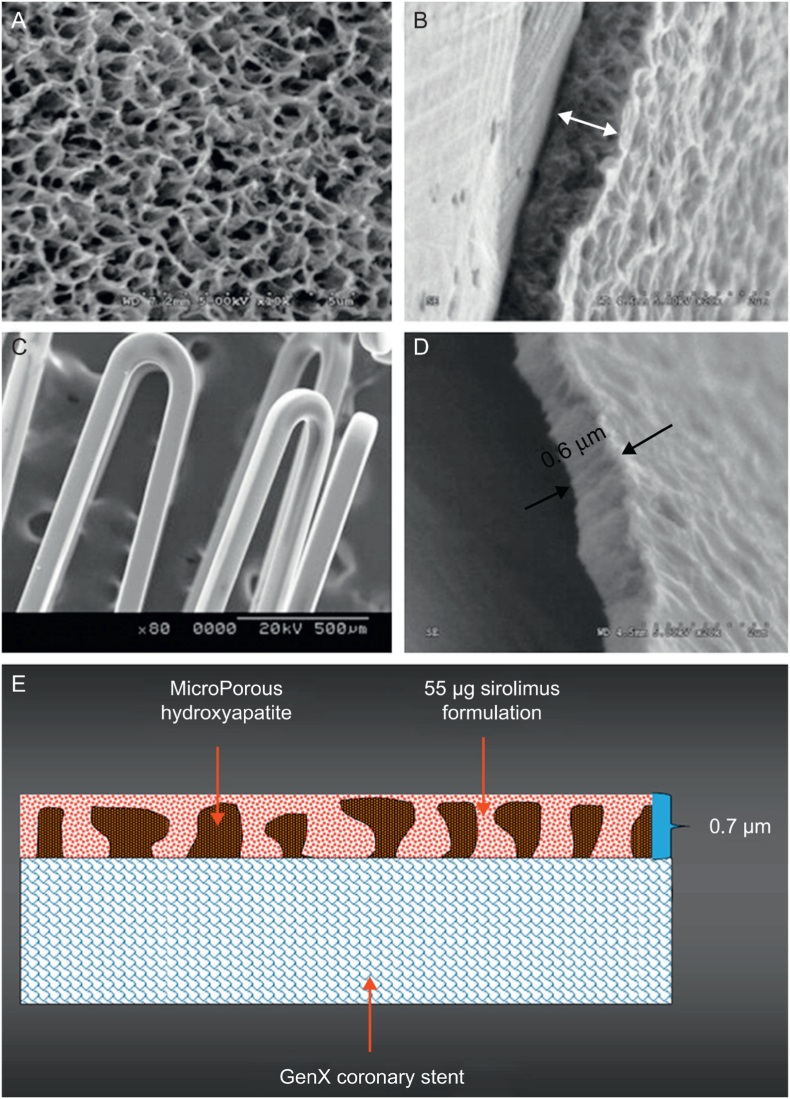
The polymer-free sirolimus-eluting stent with nanotopographical surface [63].

To improve the biosafety of organic degradation products, novel polymer-free-composite drug-eluting coatings and even unique drug-free nanotopographical technologies are introduced to eliminate the need for extra layers of a polymer [[60], [61], [62]]. The textures design and surface modification technologies enable the fabrication of specialized micro-/nano-patterns (such as pores, ridges, nanotubes, leafy structure, nanograss, nanoflakes, nanopillars, and nanowires, etc.) with or without carried drugs on thin struts to reduce the risk of blood clotting, expedite healing and improve endothelialization [61,62]. The typical VESTAsync stent (MIV Therapeutics, United States) possesses a nanoporous hydroxyapatite coating that is impregnated with sirolimus on a stainless steel stent surface (Fig. 4) [63]. However, the critical challenge for polymer-free-composite drug-eluting coatings is a sufficient level of carried drug to ensure the long-term inhibition of neointimal hyperplasia and in-stent restenosis after implantation.

By virtue of the concept of vascular healing and regeneration, there is a re-surging interest in regenerative medicine for future stent therapies. Regenerative surface modification approaches of vascular stents include stem cell secretomes [64], cell-capture coatings [65,66], mimics of endothelial products [[67], [68], [69]], soluble endothelial growth factors [70] and cell-adhesive peptides [71], etc.

**Fig. 5:**
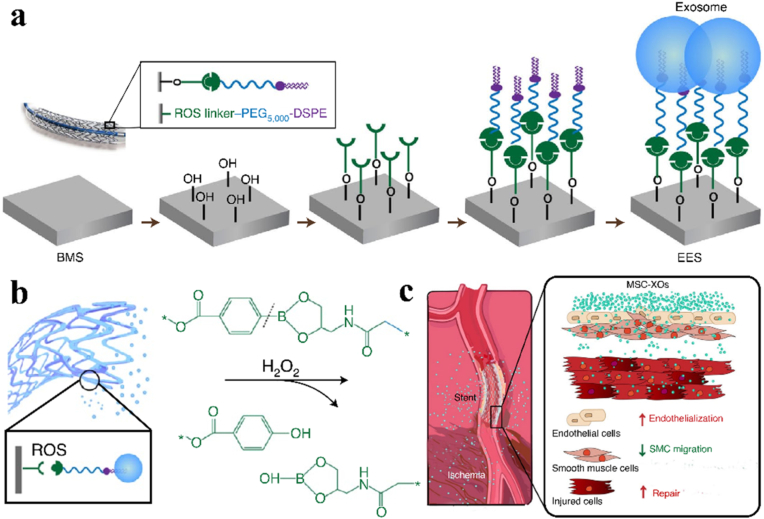
Schematic of the exosome-eluting stents (EES) for vascular healing, (a) fabrication process of EES, (b) exosome release mechanism of ROS-trigged EES, and (c) EES placement in the rat model [64].

Cardiovascular stents incorporated with cell secretomes represents a brand-new and cutting-edge development direction for regenerative stents. The exosomes of extracellular vesicles derived from mesenchymal stem cells are cognized to improve endothelial proliferation and ameliorate inflammation. Hu et al. designed and constructed a bioresponsive exosome-eluting stent (EES) for vascular healing and tissue regeneration after ischemic injury. A benzeneboronic acid pinacol ester group was utilized to trigger the stent responsive to local ROS stimuli and further release exosomes secreted from mesenchymal stem cells into the bloodstream and peripheral ischaemic tissue (Fig. 5) [64]. In the rat model, the exosome-coated stents accelerated re-endothelialization, inhibited local vascular inflammation, suppressed in-stent restenosis and promoted muscle tissue repair after implantation.

**Fig. 6:**
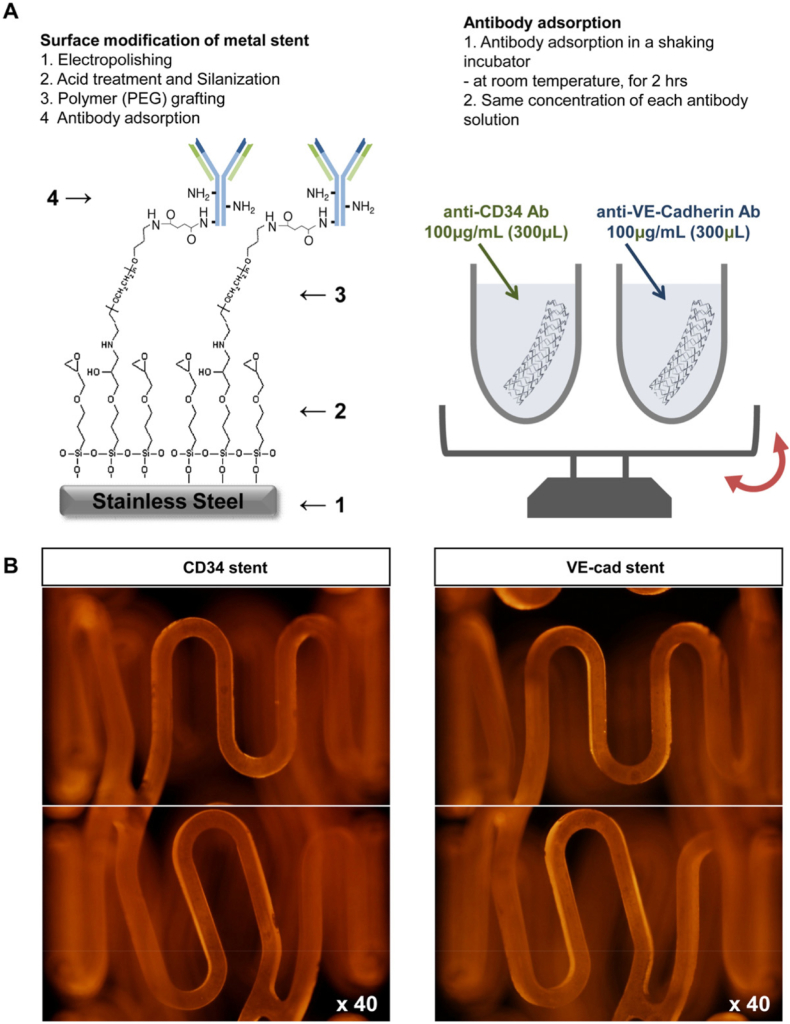
The antibody-coated cardiovascular stents. (A)The fabrication process of CD34 and VE-cad antibodies binding on stent surfaces, (B) The uniform distribution of coated antibodies was evaluated by immunofluorescence [72].

The cell-capture stent is another alternative strategy that is to utilize biomolecule coatings to capture circulating endothelial progenitor cells (EPCs) in the blood for endothelial regeneration. Various antibodies recognizing EPCs, like anti- CD34, Vascular endothelial-cadherin (VE-cad) and CD133 were loaded to the stent surface which accelerated reendothelialization and reduced the risk of in-stent restenosis. Lee et al. fabricated and compared the specific capturing EPC of anti-human VE-cadherin antibodies or anti-human CD34 antibodies on stainless steel stents. Compared with CD34 stents, VE-cad coated stents more selectively captured EPC and endothelial cells, accelerated re-endothelialization and reduced neointimal formation (Fig. 6) [72].

**Fig. 7:**
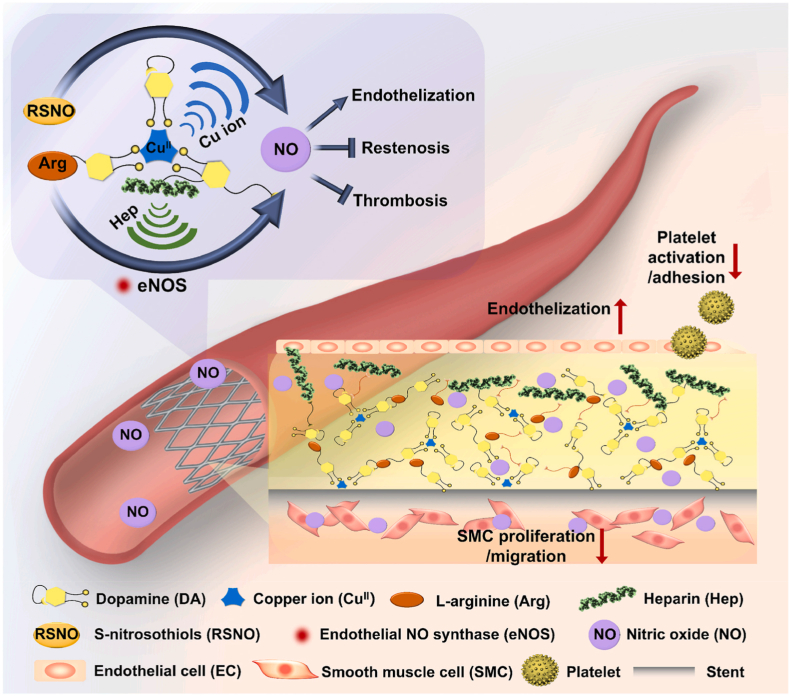
Structural and functional schematic of endothelium-mimicking, self-holding NO fueling coatings used in cardiovascular stents [73].

As an important gaseous signaling molecule in the cardiovascular system, nitric oxide (NO) plays an important role in sustaining normal physiological functions of the vessels and fine-tuning vascular homeostasis [67,68]. NO inhibits the multiplication and migration of vascular smooth muscle cells (SMCs), regulates blood pressure, preserves vascular tone, and inhibits platelet aggregation in the cardiovascular system [15]. The expression of endothelial nitric oxide synthase (eNOS), a key enzyme that primarily determines NO production, is critical for neointimal responses in cardiovascular stents [69,73]. NO-releasing and NO-generating coatings are two typical NO-producing coatings. The donors of NO-releasing coating supply NO in a relatively short period. NO-generating coatings utilize immobilize catalysts such as copper and selenium to convert endogenous NO donors. Rao et al. developed a coating based on a dopamine-copper network armed with the NO precursors L-arginine and heparin on cardiovascular stents to mimic the function of native endothelial cells (Fig. 7) [73]. The synergistic effect between Cu and L-arginine is similar to a petrol station that promotes NO production to compensate for the lack of endogenous NO donors *in vivo* [73]. The DA-Cu-Arg-Hep coating inhibited smooth muscle cell migration, promoted endothelium reconstruction, prevented stent thrombosis and restenosis, and suppressed cascading platelet adhesion [64].

**Fig. 8:**
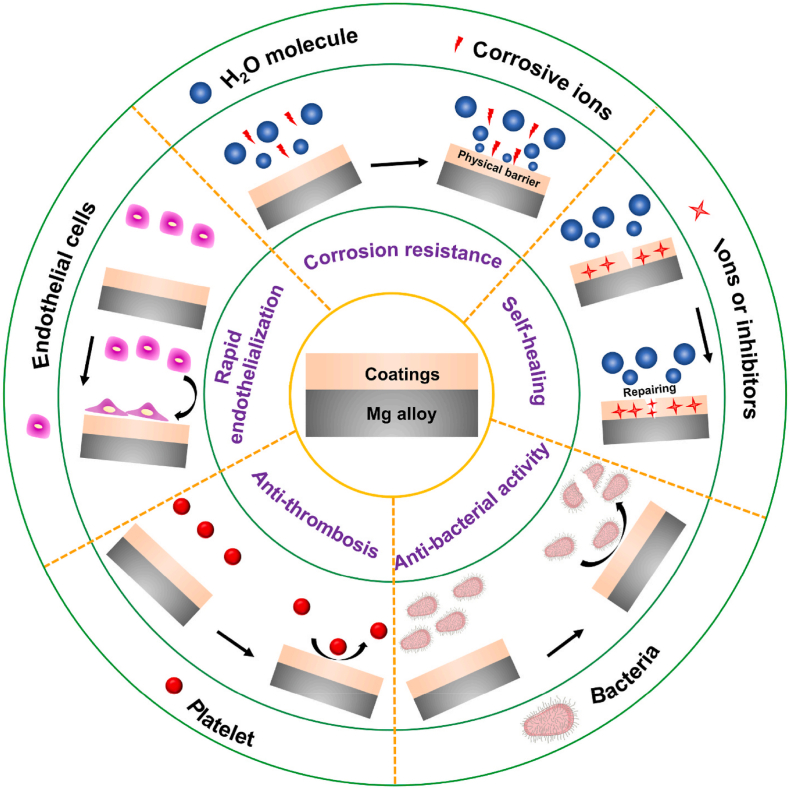
The functionalized coatings on Mg alloys for cardiovascular stents [74].

The stent has steadily evolved and renewed interest in fifth-generation drug-eluting fully-resorbable stents. The bioabsorbable metal stents, such as magnesium (Mg) alloy stents [74,75], iron (Fe) alloy stents [76,77] and zinc (Zn) alloy stents [78,79], perform outstanding mechanical strength and biocompatibility compared to polymer stents for biodegradable drug-eluting stent applications. Fe-based stents perform too slow degradation rate and raise the problem with magnetic resonance imaging (MRI) near embedded stent. Owing to suitable degradation rate and appropriate flexural strength, Zn-based stents are desirable alternatives for fully degradable metal stents. Currently, initial studies on Zn-based stents started late, it shows a lack of systematic research *in vivo* and *in vitro*. Magnesium (Mg) and its alloys are promising materials for cardiovascular stents, but they continue to be major challenges in clinical practice due to their rapid corrosion. The occurrence of thrombosis, inflammation and fast restenosis after Mg alloy stent implantation caused by their insufficient corrosion resistance usually induced stent fracture and collapse, as well as inferior endothelialization. Numerous surface modification techniques and functionalized coatings on Mg alloys for cardiovascular stents is of great significance in the promotion of the rapid endothelizlization, anti-thrombosis, self-healing and anti-bacterial activity (Fig. 8) [74].

**Fig. 9:**
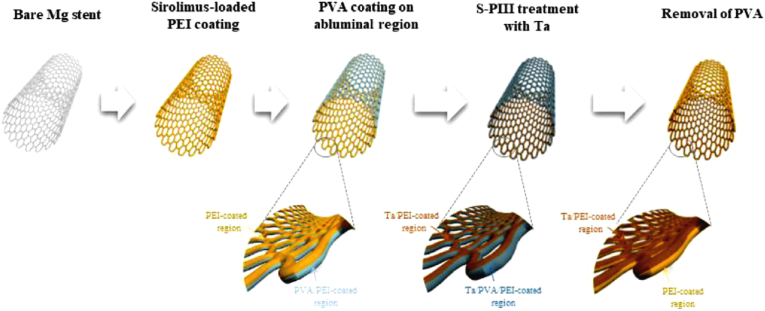
Schematic diagram of fabrication process of Ta-implanted PEI coating layer on multifunctional and biodegradable Mg vascular stents [80].

To improve sustained drug delivery ability, vascular compatibility of Mg stents, and corrosion resistance, Cheon et al. developed a new strategy for solving rapid corrosion problems of biodegradable Mg stents by combining sprayed poly (ether imide) (PEI) coating and subsequent sputtering-based plasma immersion tantalum (Ta) ion implantation (Fig. 9) [80]. PEI coating on stent demonstrated outstanding corrosion resistance and sirolimus-carrying ability for inhibition of vascular smooth muscle cell growth. A 20 nm Ta-implanted layer on the topmost PEI surface can modify wettability and effectively control sirolimus release rate, which further prevented direct exposure of the sirolimus to the surface adhered endothelial cells and platelet activation rates for in-stent restenosis and thrombosis.

**Fig. 10:**
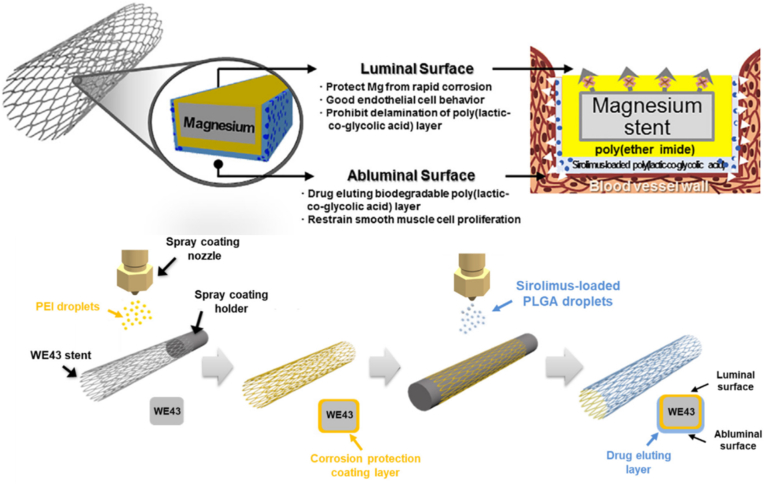
Schematic diagrams showing the sprayed asymmetric surface coating with PEI and PLGA/PEI layers on the WE43 stent [81].

Large-scale surface functionalization by spray deposition of multilayers is an efficient measure in regulating both degradation behavior and biofunction of Mg-based stents. Jang et al. developed a novel asymmetric coating using a continuous spraying method [70] to deposit a polyetherimide (PEI) single-layer coating over the inner surface of the stents and a sirolimus-loaded poly (lactic-co-ethylene glycolic acid) (PLGA)/PEI bilayer coating over the outer surface (Fig. 10) [81]. Excellent adhesion of the PEI coating to the surface of WE43 significantly improved the corrosion resistance and *in vitro* endothelial cell compatibility of WE43. The PLGA/PEI bilayer coating had a stable drug release surface morphology and a low sirolimus release rate.

## 3 Digestive tract stents

Currently, stents are used for both radical and palliative treatment of malignant obstruction of the GI tract, and nowadays they are also used in benign cases such as perforation, stenosis or fistulae. The development of these drug-eluting stents not only provides patients with palliative care, but also offers possible treatment options of anti-tumor due to the release of antidepressants (idionine-125) [82]. For example, hepatobiliary diseases are among the most frequent diseases that pose a serious threat to human health and life today. As an important palliative treatment, transhepatic biliary stenting effectively relieves jaundice, creates the conditions for further radiotherapy, chemotherapy, and other treatments, prolongs survival, and improves life quality [[83], [84], [85], [86]]. Biliary stent placement is typically applied to prevent preoperative acute biliary obstruction during endoscopic retrograde cholangiopancreatography (ERCP). This stent is an implantable device made of the materials like silicone, cobalt-chrome, and nitinol.

Implantation of biliary stents affects bile duct dynamics, leading to bile concentration and triggering cholestasis [87]. Bile concentrate stimulates inflammatory changes in the biliary mucosa, facilitating the deposition of leukocytes, fibrin and shed epithelial cells [88,89]. It can also increase the concentrations of unconjugated calcium, bilirubin, bile acids, and glycoproteins, promoting bile pigment stone formation [[90], [91], [92]]. In addition, after biliary stent implantation, the loss of sphincter function and the decreased bile duct pressure can induce bacterial reflux from the duodenum into the bile ducts, in turn leading to biliary tract infections and stent obstruction [88,89]. However, current stents only provide biliary drainage and do not have antitumor activity. Over time, even metal stents may become occluded due to the tumor overgrowth or ingrowth, tissue proliferation, and cholestasis, leading to progressive biliary obstruction and liver failure [93,94].

Esophageal cancer is ranked as the eighth most common cancer and the sixth most common cause of cancer deaths worldwide [95]. The most significant complaint of these patients is the tumor-related dysphagia. Placing a self-expanding metal stent is considered the best option for symptomatic relief [82]. In this respect, drug-coated biliary stents, drug-coated esophageal stents, or drug-coated intestinal stents are being developed to meet the clinical needs of the digestive system.

### 3.1 Drug-coated biliary stents

These special stents are usually designed to create channels through the bile ducts to prevent body fluids from clogging or blocking them. However, accidental stent occlusion could result in jaundice, recurrence of cholangitis, or even sepsis, which could be life-threatening if left untreated [7,22]. Patients will suffer stent removal and re-implantation. The interface behavior of the stent material in the body fluid microenvironment significantly affects the service life. Early stent occlusion (less than one month) mostly occurs due to blood clots, positioning error, debris, or mucus from mucin-producing tumors. For late stent occlusion (more than one month after placement), the generally accepted theory is that both bacterial biofilms and cholestasis play important roles [22].

**Fig. 11:**
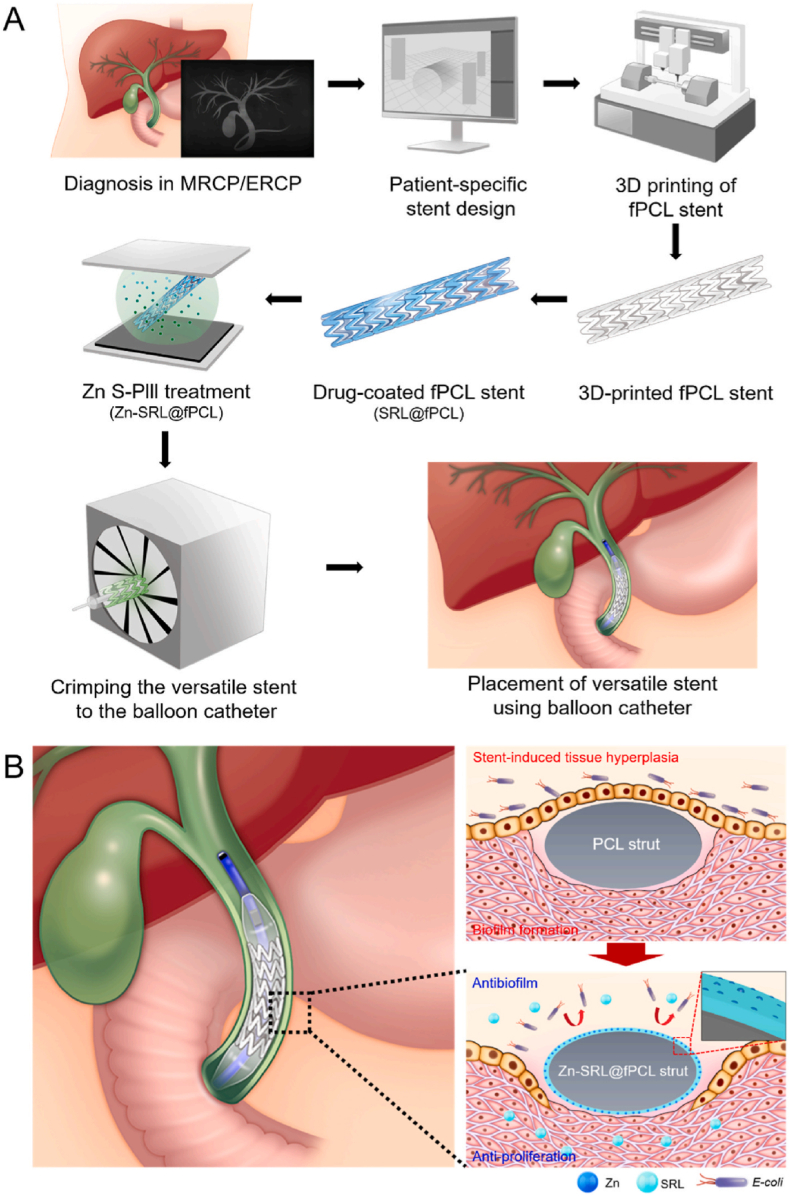
(a) Preparation process of degradable multifunctional PCL (fPCL) biliary stent. (b) A sirolimus (SRL)/PLLA coating embedded with the Zn ions on stent surface effectively suppress bacterial biofilm formation and fibroblast cells proliferation [100].

Cholestasis that causes stent blockage consists mainly of calcium bilirubinate and calcium palmitate crystals formed by bacterial enzymes [22]. It is well known that the adsorption process of key proteins such as vitronectin, fibronectin, laminin and collagen on the surface of the scaffold plays an important role in the formation of sludge [96]. The micro-organism accumulation of aerobic bacterial species (gram-positive *Enterococcus* species, and gram-negative *Escherichia coli* and *Klebsiella* species), anaerobic bacterial species (*Clostridium* species), biofilm, and fungi formation are known to initiate stent blockage. Sludge subsequently coats the biological film, causing rapid narrowing of the inner diameter of the biliary stent [97]. Hydrophilic, antibacterial, or drug-containing coatings can reduce sludge formation and inhibit bacterial colonization. There have been attempts to combine antibiotics with choleretic agents such as ursodeoxycholic acid [98,99] or terpenes [99] to produce a synergistic effect. Lee et al. reported a versatile biodegradable biliary stent with surface-functionalized drug coating for antibiofilm formation. The sirolimus-containing polymer coating embedded with zinc (Zn) ions via the sputtering-based plasma immersion ion implantation treatment (Fig. 11) on 3D-printed fPCL stents. The viability and biofilm density of adhered bacteria on the Zn-SRL@fPCL stents significantly decreased than those covered on the surfaces of PCL and fPCL stents. The sustained release of Zn ions as a nonspecific biocide from the drug coating exerted a broad-spectrum antimicrobial effect against *E. coli* and *S. aureus*, indicating that Zn-SRL@fPCL stent may be a promising antibacterial strategy for biofilm induced stent blockage [100].

For bile duct malignancies, self-expanding metal stents are superior to plastic stents for palliative care. However, over time, even metal stents can become occluded due to overgrowth or ingrowth. Conventional metallic biliary stents offer only mechanical relief and have no anti-tumor effect. Local administration of chemotherapeutic agents maximizes the concentration of the drug in the tumor microenvironment and reduces adverse effects and non-target organ toxicity associated with systemic exposure [101]. Therefore, stents for topical delivery of biochemical agents, such as drug-eluting stents, are a promising approach for the treatment of malignant biliary obstruction.

**Fig. 12:**
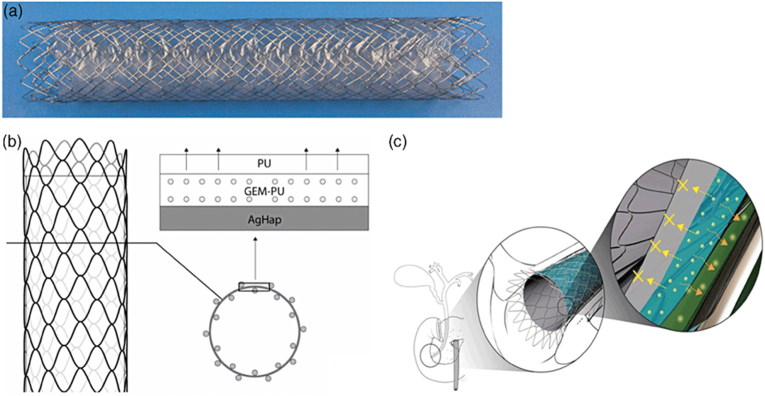
Gemcitabine-incorporated membrane containing drug partially covering a self-expanding metal stent [103].

More effective than other chemotherapeutic agents, gemcitabine (GEM) has been reported for unresectable pancreatic and biliary tract cancers [102]. Park et al. reported a novel self-expanding metallic biliary stent eluting GEM [90]. The functional membrane of GEM-eluting stents comprises three layers: a polyurethane film at the top, a polyurethane film containing GEM in the middle, and the bottom silicon membrane containing silver-hydroxyapatite (Fig. 12) [103]. It is safe to use GEM-eluting stents in normal bile ducts. Stents containing 10 percent GEM create minor histological changes within the stent-adjacent and segment tissues [103].

**Fig. 13:**
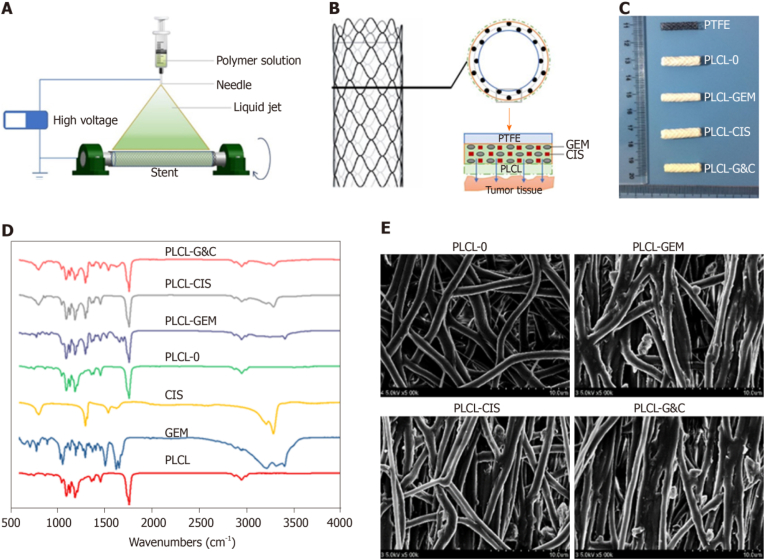
Manufacture and characteristics of drug eluting stents for the biliary tract [104].

Currently, gemcitabine (GEM) and cisplatin (CIS) are the standard chemotherapy regimen for cholangiocarcinoma therapy [102]. The two drugs improve survival rate without apparent toxicity. Wan et al. applied double chemotherapeutic agents in a drug-eluting stent, which has some design improvements over previous stents, for the palliative treatment of extrahepatic biliary cancer [104]. An innovative GEM and CIS-eluting nickel-titanium biliary stent was designed and fabricated using the electrostatic spinning method (Fig. 13) [104]. The stent was covered with a polytetrafluoroethylene (PTFE) membrane, which was engineered as an internal blocker to prevent drug loss in the bile stream and to ensure maximum drug delivery to tumor tissue. A PLCL membrane was further fabricated as the outer layer of the drug-loading stent to avoid the burst release of the drug. GEM and CIS-loaded films significantly inhibited the growth of EGI-1 cholangiocarcinoma cells and animal tumor xenograft model. *In vivo* experiments confirmed that stents coated with GEM and CIS-eluting nanofibrous membranes have favorable safety and biocompatibility in native porcine bile ducts.

**Fig. 14:**
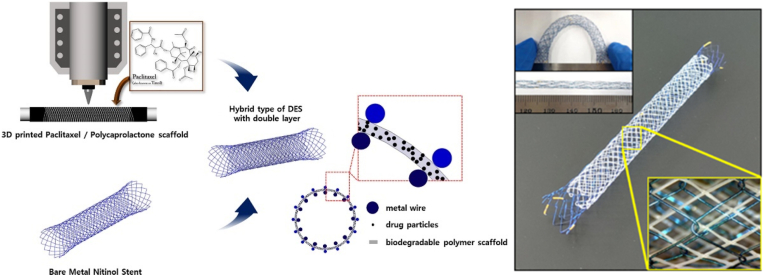
Design of a schematic for the construction of a Bi-Layered advanced stent with 3D printed PTX/PCL stents and self-expanding metal stents [105].

Paclitaxel (trade name Taxol) is a well-known anti-cancer drug [33]. To prevent stent occlusion resulting from tumor overgrowth and ingrowth, Park et al. reported a novel self-expanding metallic stent using 3D printing technique to fabricate tubular polycaprolactone (PCL) stents with varying concentrations of paclitaxel (PTX) (Fig. 14) [105]. The tubular stents were physically assembled between the layers of the self-expanding metallic stents to form an advanced stent.

**Fig. 15:**
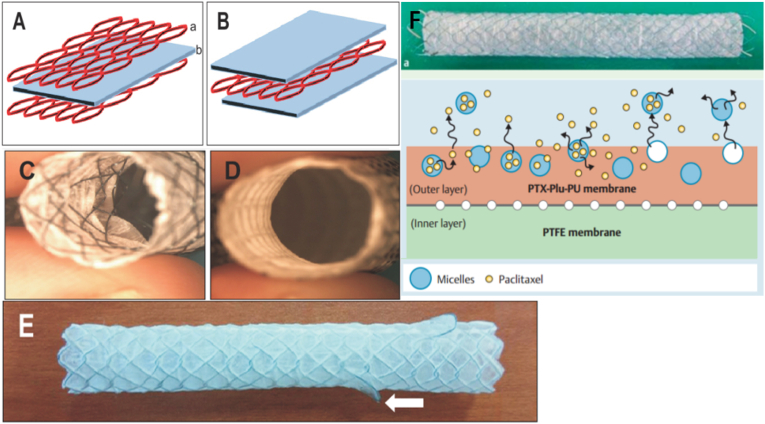
Structure of modified metallic stents with paclitaxel-impregnated membranes (MSCPM-III) [107].

Sodium sebacate (SC) is a drug known to facilitate the absorption of several drugs [106], such as insulin, ampicillin, 5-fluorouracil, and low molecular weight heparin. The third-generation MSCPM-III stent has a bilayer structure with an inner layer composed of bile acid-resistant polytetrafluoroethylene (PTFE) and an outer layer composed of polyurethane (PU). Lee et al. reported a metal scaffold MSCPM-III coated with SC binding membrane (MSCPM) and paclitaxel (PTX) (Fig. 15) [107]. SC was added to increase the ability of PTX eluting from the MSCPM-III stent to penetrate cancerous tissues [108]. The stent coat consists of PTX-SC-PU polymer coated with tetrahydrofuran (THF) solution. According to the intensity of reactive inflammation and fibrosis, metallic biliary stents MSCPM-III containing paclitaxel and sodium decanoate seem to be safe in pig bile ducts.

### 3.2 Drug-coated esophageal stents

Esophageal cancer (EC) is the sixth leading cause of death from cancer globally, with about a 15 percent five-year survival rate [109]. Patients with EC are often identified at an advanced stage, making them unfavorable to undergo surgical resection. Palliative treatments provide relief of symptoms and cancer pain, but cannot effectively suppress tumor cells. Esophageal stenting is a proven treatment option for relieving esophageal strictures and inhibiting tissue proliferation induced by stenting [[110], [111], [112]]. In the clinic, radiotherapy and chemotherapy commonly cause acute side effects. Antineoplastic drug/esophageal stent combinations provide relief of local chemotherapy for cancer and malignant esophageal strictures. Recently, it appears that the most effective treatment for esophageal cancer pertains to the use of drug-coated stents for local drug delivery and anticancer therapy, which results in immediate relief of esophageal strictures and provides further chemotherapeutic effects at the tumor site to inhibit tumor growth while minimizing side effects. Controlling drug release and promoting sustained penetration of the drug into the deeper tissues following placement of the combination at the site of a malignant esophageal stricture is essential for the effective treatment of localized tumors.

**Fig. 16:**
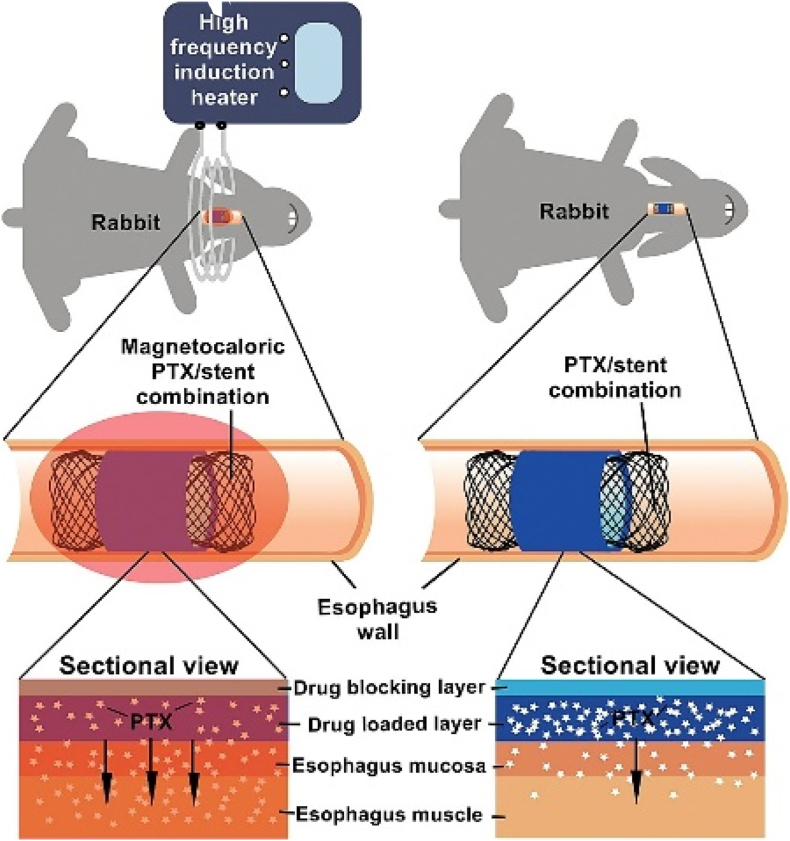
Schematic diagram of PTX/nitinol stent [113].

Guo et al. reported a new method for modifying the surface of esophageal stent materials with temperature-responsive phase-change 1-hexadecanol for magnetic delivery of drugs. Magnetic nitinol stents were coated with a bilayer film, in which one layer of ethylene vinyl acetate copolymer (EVA) acted as a drug barrier, and the other layer of EVA contained 10 % paclitaxel (PTX) and 30 % temperature-sensitive phase-change fatty alcohols (1-octadecanol, 1-tetradecanol, or 1-hexadecanol) (Fig. 16) [113]. PTX can be released magnetically and penetrate the esophageal wall efficiently from the PTX/Nitinol stent combination. Pathology data demonstrated the biocompatibility and safety of the combination after placement in rabbit esophagus, even in the presence of alternating electromagnetic fields.

**Fig. 17:**
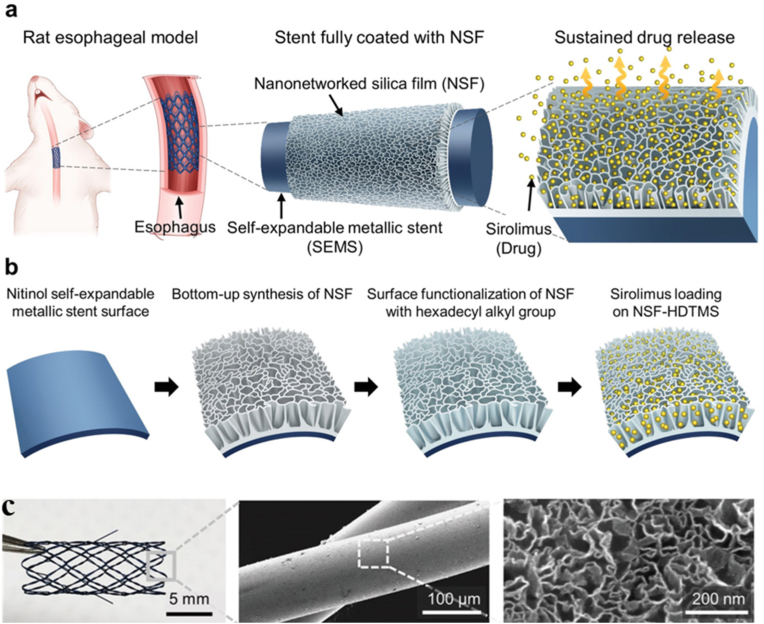
Scheme and design of polymer-free drug-eluting stents (DES) for tissue proliferation inhibition [114].

The application of self-expanding metal stents (SEMS) is highly restricted by tissue proliferation on the stent surface, which usually triggers stent obstruction. Tissue proliferation due to stent placement is a proliferative response to mechanical damage to the esophageal wall caused by the stent [31,32]. Local heating significantly inhibits stent-induced tissue proliferation [31]. Lee et al. developed a functional 3D nano-networked silicon dioxide film (NSF) that can be used as the platform for drug delivery across the entire surface of self-expanding metallic stents (SEMS) [114]. The entire scaffold was covered with NSF and the drug was packed into hydrophobically modified NSF nano-networks (Fig. 17) [114]. NSF with a porous network was synthesized on nickel-titanium stents using top-down strategies with triethanolamine (TEA), sodium salicylate (NaSal), cetyltrimethylammonium chloride (CTAC), and tetraethyl orthosilicate (TEOS) after continuous assembly and deposition. Then, the sirolimus (SRL) was loaded onto functionalized NSF film with hexadecyltrimethoxysilane (HDTMS). In rat esophagus, SRL-loaded NSF SEMS inhibited stent-induced tissue proliferation significantly more than control SEMS.

**Fig. 18:**
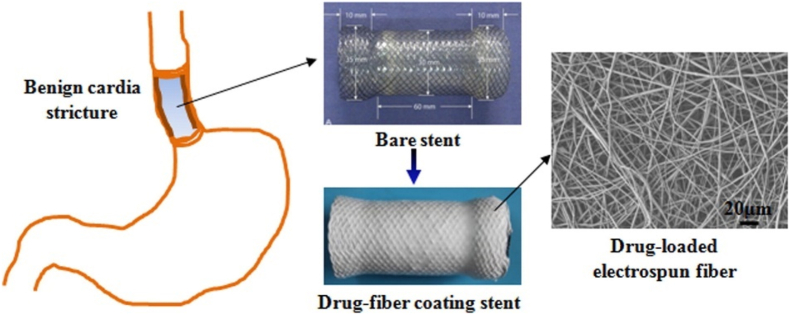
Manufacture of a stent coated with a paclitaxel-containing electrospun PCL fiber membrane by attaching a bare stent to a rotor as a collection device for benign cardia stenosis treatment [111].

Tissue proliferation and inflammatory response within the stent after stent placement have a significant impact on stent retention time, scar formation, and long-term recurrence rates. In addition, esophageal endothelial tissue is highly susceptible to tearing when the stent is removed from the esophageal tissue because it is difficult to detach the stent from the esophageal tissue, which consequently gives rise to secondary injury [115,116]. Absorbable and biodegradable esophageal stent materials are being developed to address this issue. Zhu et al. showed the development of a biodegradable electrospun drug fiber-coated scaffold (DFCS) for inhibiting inflammation and scar formation. The rotating collection method was used to deposit electrospun paclitaxel/poly(e-caprolactone) (PCL) fibers on a bare stent (Fig. 18) [111]. Paclitaxel was released mainly via drug diffusion. DFCS showed a marked reduction in inflammation and collagen fibrillar hyperplasia and was easily extracted from the esophageal portion with little or no damage to the tissues in the dog model.

**Fig. 19:**
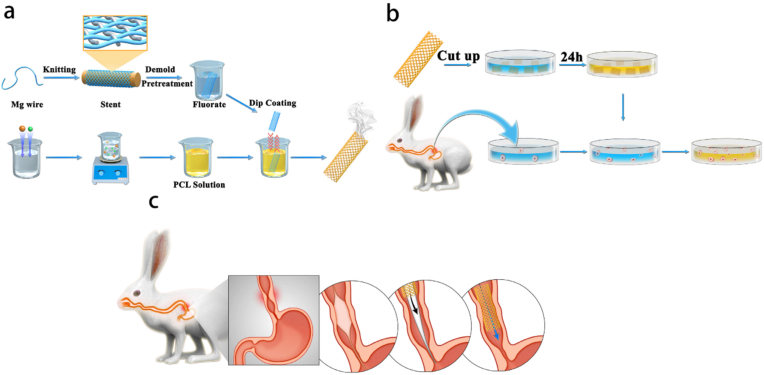
Primary procedures and methods for (a) production of PLGA-coated drug-eluting magnesium stents, (b) *in vitro* degradation, drug release and cytotoxicity assays, and (c) stent implantation [112].

In the last decades, one of the most revolutionary research topics has been the development of biodegradable and resorbable stents. Biodegradable stents can be gradually degraded, thus avoiding secondary removal through endoscopic procedures. Teng et al. reported a biodegradable woven scaffold based on magnesium (Mg) with a surface coated with paclitaxel-containing (PTX) poly (lactic-co-glycolic acid) (PLGA) (Fig. 19) [112]. With the stent degradation, the PTX is continuously released, which effectively enhances fibroblast apoptosis. In the New Zealand rabbit model, the scaffold reduced the number of inflammatory cell infiltration and fibrous tissue. With these results, PTX-PLGA-coated magnesium stents are promising as a safe and effective treatment for benign esophageal strictures.

### 3.3 Drug-coated intestinal stents

Bowel stenosis is a frequent surgical condition and a consequence of certain diseases or surgeries. Bowel stents can be used to treat not only malignant intestinal stenosis such as colon cancer obstruction but also benign intestinal stenosis such as colonic fistula, anastomotic fistula, perforation, and inflammatory bowel stenosis [[117], [118], [119]]. 7%–29 % of colorectal cancer patients may develop bowel obstruction and stenosis [120,121]. Restenosis of intestinal stents due to tumor infiltration and growth remains a major problem. Therefore, drug loading and elution on intestinal stents that restore luminal function and have intraluminal anti-tumor capabilities are essential to overcome these limitations.

**Fig. 20:**
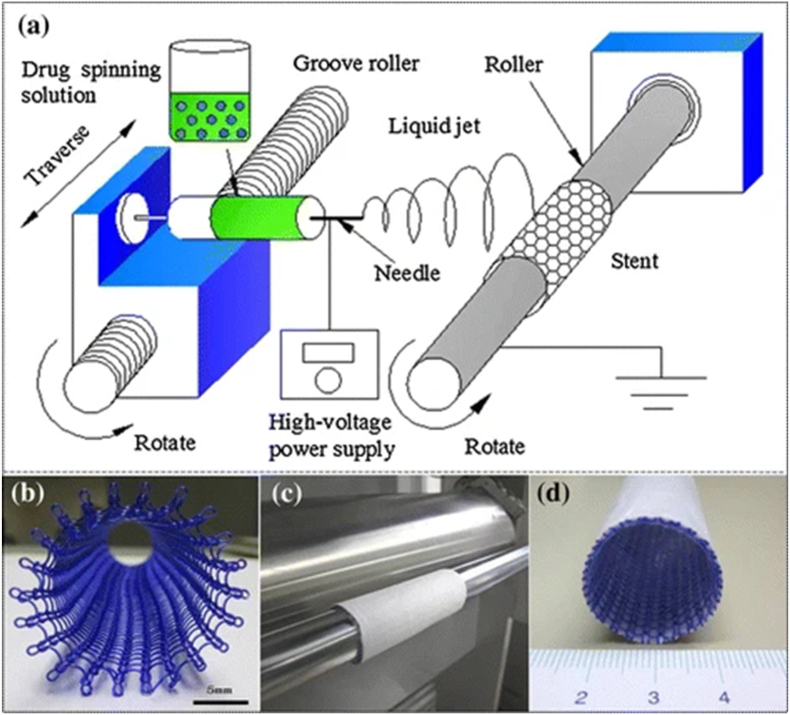
Stent coating system with seamless 5-FU PLA film coated onto the stent surface [122].

Lan et al. reported a method of electrospinning 5-fluorouracil (5-FU)-loaded poly-L-lactic acid membranes onto weft-knitted polydioxanone stents to relieve intestinal obstruction and stenosis (Fig. 20) [122]. The results showed that at suitable drug loading doses like 6.4 % or 12.8 %, the drug film had a significant antitumor effect, which was attributed to the sustained release of 5-FU from the drug film. In the future, surface-modified stents hold great promise for the clinical treatment of bowel cancer.

## 4 Respiratory stents

Airway stenosis (AS) is generally classified into structural stenosis, which is the narrowing of the airway lumen caused by the growth and compression of various primary benign and malignant tumors and metastatic malignant tumors, and functional stenosis, which occurs most often in tracheal chondromalacia [123]. Tracheal stents and tracheobronchial stents have been explored for airway obstruction and acute respiratory failure. As the most common functional substitute for tracheobronchial tubes, they can rapidly restore narrowed airway patency and relieve dyspnea symptoms [124].

Complications associated with airway stenting mainly include infection or secondary pneumonia, granulation formation, sputum stasis, and stent migration or malposition [[125], [126], [127], [128], [129]]. Airway stent placement has become an important therapeutic strategy with the continuous development of new materials and fabrication techniques. Stents commonly used in clinical practice mainly include nickel-titanium memory alloy and silicone stents. Stents can be successfully removed endoscopically if complications arise. However, stent abrasion of the airway mucosa induced inflammatory repair and excessive granulation tissue hyperplasia have significantly affected the airway restenosis in clinical therapy. Therefore, suitable stents, biodegradable materials, and appropriate surface modification processing may reduce stent restenosis and stent complications.

**Fig. 21:**
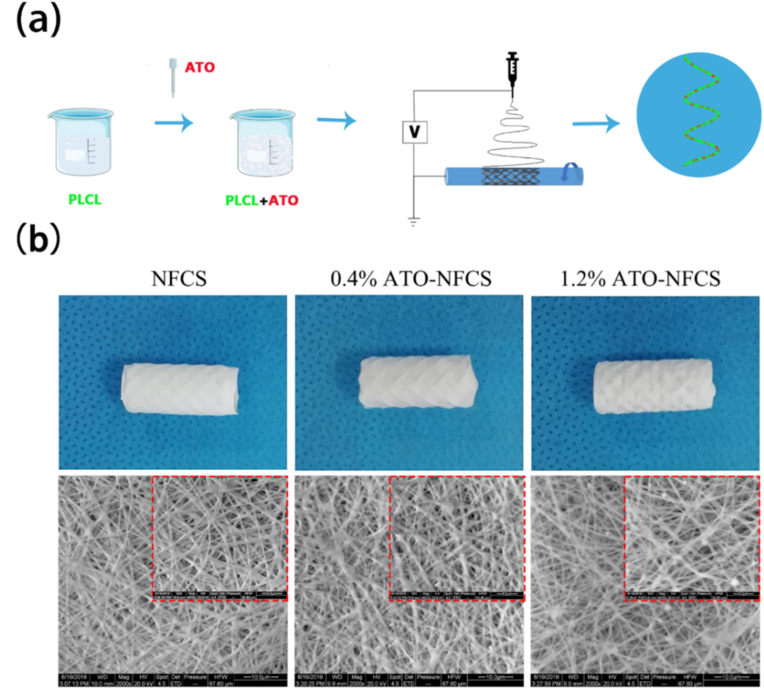
Schematic representation of the manufacturing and physicochemical properties of ATO-NFCS [130].

Granulation tissue hyperplasia associated with stents is a major complication limiting the use of airway stents. Bare stents cause exposure to the airway wall mechanically (friction and contact pressure), resulting in adverse granulation proliferation and subsequent restenosis. Innovations in tracheal stents have been supported by recent advances in materials science. To inhibit granulation tissue proliferation and reduce restenosis after the placement of a nickel-titanium alloy stent, Li et al. reported a self-expanding metallic scaffold covered with arsenic trioxide-eluting electrospun nanofibers (ATO-NFCS) (Fig. 21) [130]. Poly(L-lactide-co-caprolactone) (PLCL) was chosen as the carrier polymer and ATO as the antiproliferative drug to prepare nanofibrous membranes by electrostatic spinning. The ATO release from the drug-carrying polymer effectively inhibited proliferation of human embryonic pulmonary fibroblasts, airway smooth muscle cells and bronchial epithelial cells. In a rabbit model, their results showed that ATO-NFCS was safe *in vivo* and effective in inhibiting granulation tissue formation.

**Fig. 22:**
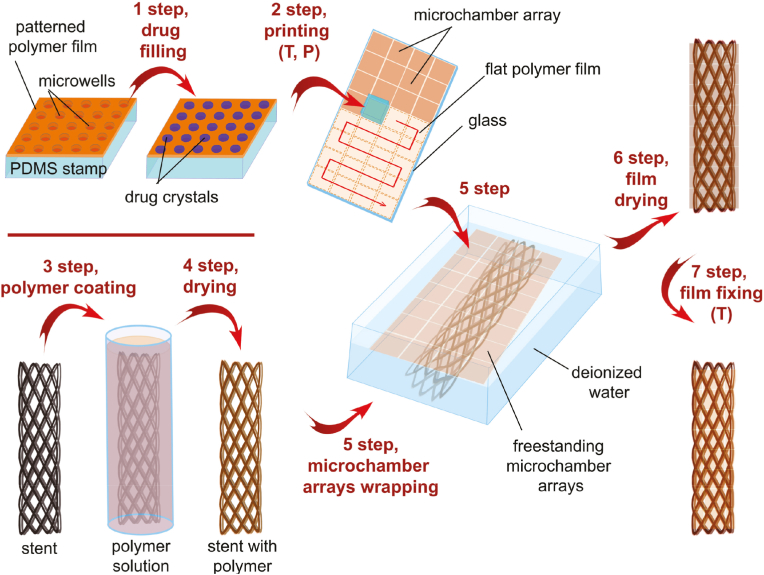
A general scheme for surface functionalization of tracheal stents using biodegradable microcavity arrays containing anti-inflammatory agents [40].

Covered stents and packed steroid drugs block proliferation granulation and tumor overgrowth. However, the cover hampers sputum expectoration and increases respiratory infection. Sindeeva et al. investigated the use of patterned drug-eluting coatings for biodegradable trachea stents based on polylactic acid, polylactic acid ethylene vinyl acetate, and polylactic acid propylene ester, which reduce particle formation (Fig. 22) [40]. Microchamber arrays on the polymer film surface of the modified nitinol stent are suitable for loading drugs and keeping high-molecular-weight cargo in these microchambers. The results of the rabbit experiments showed that the use of PLA and PLGA coatings was effective in inhibiting granulation tissue.

**Fig. 23:**
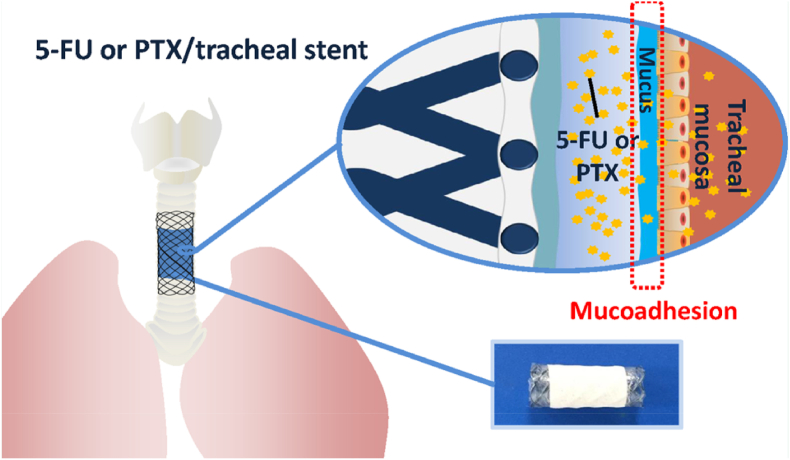
Anti-migration nitinol tracheal stent with paclitaxel and 5-fluorouracil [131].

Displacement of the stent is one of the common complications of tracheal stent failure. Antineoplastic drug/tracheal stent combinations provide prompt relief of dyspnea due to tracheal stenosis and local treatment of malignant space-occupying lesions or tumors. In order to achieve more effective treatment by preventing stent migration, Jin et al. described a nitinol tracheal stent (TS) that prevents migration by depositing a high dose of 5-fluorouracil or paclitaxel (5-FU/TS or PTX/TS) through a surface coating. The bilayer film consists of a drug-loaded layer with Carbopol 974P as a matrix for mucus adhesion and a blank Carbopol 974P layer (Fig. 23) [131]. The anti-migration ability of 5-FU/TS or PTX/TS is governed by the composition of the bilayer. 5-FU and PTX release is dominated by the relaxation and diffusion mechanisms, respectively. Following PTX30/TS implantation in the trachea of rabbits, an inflammatory response was observed, which gradually resolved during the follow-up period.

## 5 Reproductive system stents

Urological stents and catheters are widely used as medical devices for internal urinary drainage and in oncology patients to relieve extrinsic compression and obstructive uropathy. Currently, metallic and plastic stents are primarily used in urology. Compared to plastic stents, metal stents have stronger compressive strength and can provide more proper drainage [132,133].

**Fig. 24:**
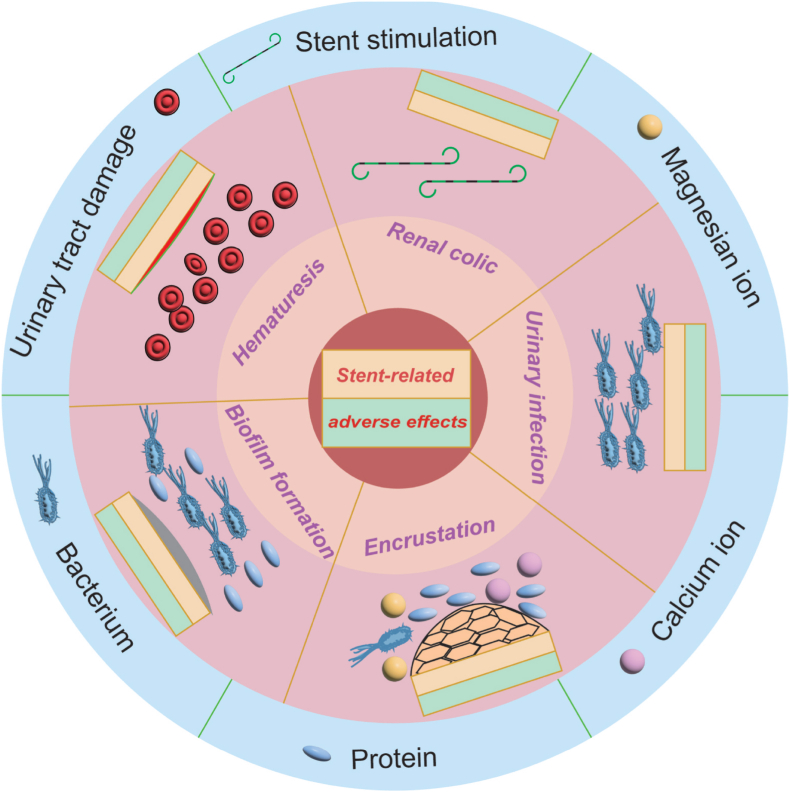
Frequent adverse reactions and complications of ureteric stent insertion [134].

Unfortunately, complications and side effects caused by urinary stents are usually related to stent migration, urinary tract infections, hematuria, abdominal discomfort, and stent crustation (Fig. 24) [134]. Ureteral stents are associated with significant adverse effects in more than 80 % of patients, severely deteriorating their life quality. Ureteral or urethral peristalsis may be one of the main causes of urinary metal stent failure. This peristalsis leads to greater rates of displacement and the development of urethral hyperplasia, which may result in obstruction. Another side effect is the frequent contamination of urine with bacteria, which has a 100 % chance of forming a biofilm on the surface of the stent, leading to encrustation that may cause obstruction. The urinary stents provide an optimal surface for bacterial attachment and biofilm formation, leading to difficult-to-treat infections.

Ureteral stents are usually surface modified to help solve the above problems. The coated stents can be classified into antimicrobial coatings, anti-crusting coatings, and hydrophilic coatings. Drug-eluting stents can be classified into antispasmodic and analgesic drug-eluting, antimicrobial drug-eluting, anti-tumor drug-eluting and anti-ureteral stenosis drug-eluting. Ureteral stent surface modification not only enhances the treatment of certain urological diseases, but also decreases the complications associated with ureteral stents.

**Fig. 25:**
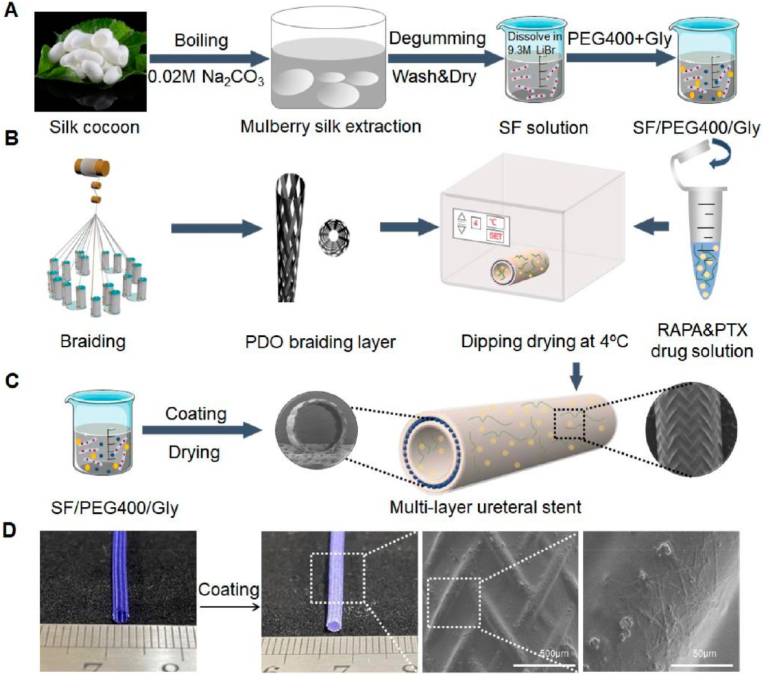
Schematic diagram of the fabrication process of drug-carrying ureteral stents [135].

Ureteral strictures and upper tract urothelial carcinomas are the most common diseases that affect the ureter. Ureteral stricture is characterized by ureter narrowing and usually occurs with chronic obstruction of the upper urinary tract. If left untreated, urine will stagnate in the upper urinary tract and renal pelvis, eventually leading to complications like urinary tract infection, hydronephrosis, even kidney failure and kidney damage [133]. Duan et al. developed an antistenosis drug-eluting poly(p-dioxanone) (PDO) ureteral stent. Rapamycin and paclitaxel-loaded silk fibroin (SF) solution was deposited on the inner and outer layers of the biodegradable PDO stent (Fig. 25) [135]. In rat experiments, drug-loaded ureteral stents showed desirable biocompatibility and inhibited fibroblast overgrowth and collagen fiber deposition. Dual-drug degradable ureteral stents showed the potential to treat ureteral strictures and avoid complications such as inflammation and strictures.

**Fig. 26:**
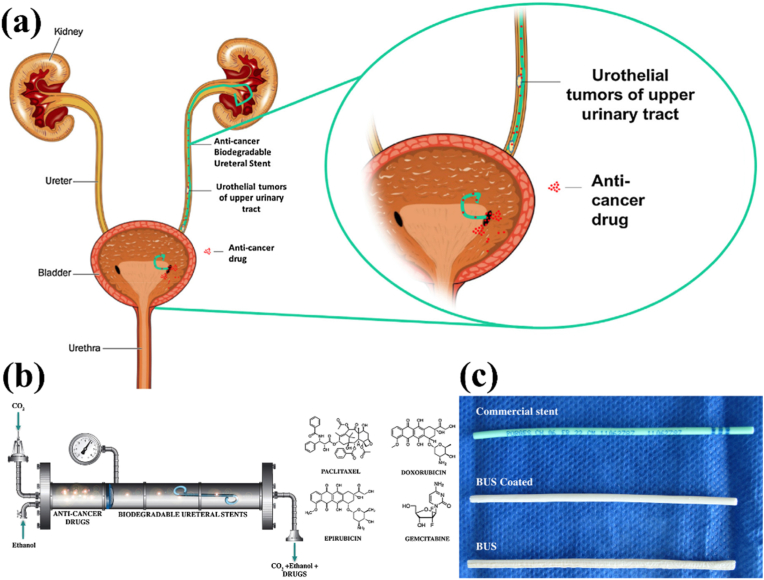
Schematic diagram of the fabrication process of anti-cancer drug-carrying ureteral stents [136].

Urothelial carcinoma is a common malignancy of the bladder and is highly susceptible to progression to bladder cancer and extravesical transitional cell carcinoma. Upper urinary tract uroepithelial cancer (UTUC) has not been studied as extensively as bladder cancer. To improve drug retention, Barros et al. utilized supercritical fluid CO_2_ to develop a biodegradable ureteral stent (BUS) impregnated with four anticancer drugs (epirubicin, paclitaxel, gemcitabine, and doxorubicin) (Fig. 26) [136]. Anti-cancer drugs can be sustainably released in artificial urine. The four anticancer drugs were impregnated at higher levels and released more slowly than commercial stents. The results suggest that dipped biodegradable ureteral stents can be used as carriers of anticancer drugs and have the potential to be an efficient and sustained intravesical drug delivery system for the management of epithelial cancers of the upper urinary tract.

**Fig. 27:**
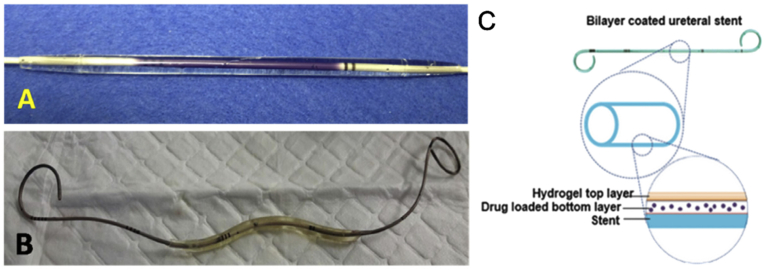
(a) Double-layer coated stent and (b) coated stent taken out after the study. (c) Schematic of a double-layer coated ureteral stent [137].

There is currently strong interest in new drug administration strategies to improve treatments and therapies in the upper urinary tract. Lim et al. demonstrated the development of a bilayer swellable drug-eluting ureteral stent (BSDEUS), which was sprayed with a polymeric drug layer containing polylactic acid-coco-lactone (PLC), which was overlaid with a swellable polyethylene glycol diacrylate (PEGDA) hydrogel (Fig. 27) [137]. BSDEUS expands to co-adsorb with the wall of the ureter, while PEGDA potentiates local drug delivery to the hyperpermeable uroepithelium to treat uroepithelial disease. In an animal experiment, a double-coated stent evidenced that the swollen hydrogel could co-adsorb with the urinary tract mucosa to achieve localized drug control and delivery to the target tissue. BSDEUS caused no hydronephrosis or systemic toxicity during the 1-day duration of the indwelling stent. There are potential applications for hydrogel-expandable drug-eluting polymer coatings for drug-based urological therapy.

**Fig. 28:**
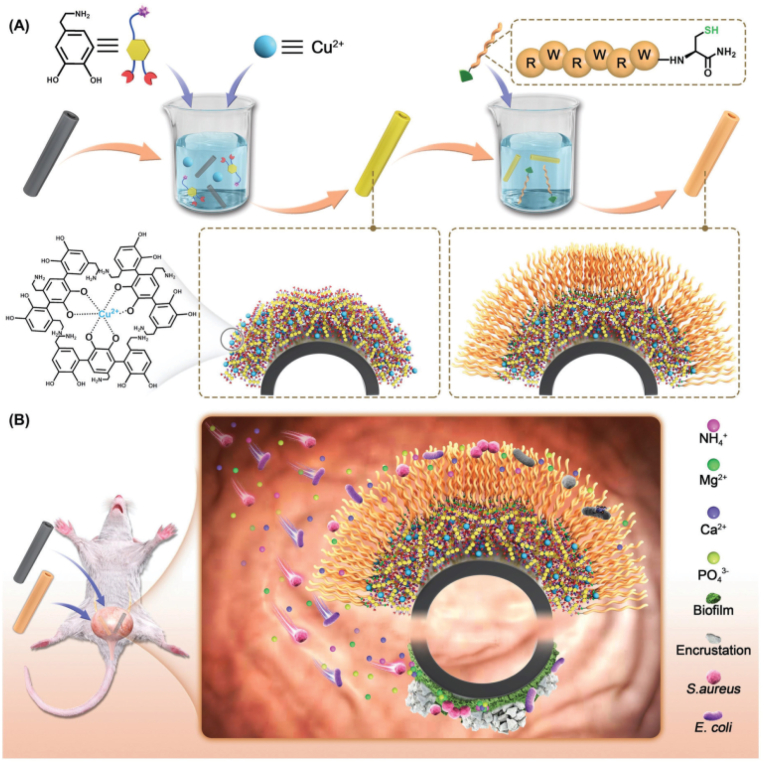
Schematic representation of anti-crusting strategies and bio-inspired antimicrobial. (A) Molecular structures of DOPA and AMP and the synthesis process of bio-inspired antimicrobial coatings. (B) Anti-crusting effect of bio-inspired antimicrobial coatings [138].

Stent-associated urinary tract infections (UTIs) are one of the most frequent nosocomial infections and are primarily associated with bacterial adherence and colonization of indwelling stents. When used, the regulatory membrane proteins on the stent further adsorb proteins, even bacteria and urinary salts. Bacteria and biofilms increase the urine pH, leading to Ca^2+^ and Mg^2+^ deposition and ultimately stent blockage. To date, drug elution techniques have been the most prospective in inhibiting the adhesion of bacteria and biofilm formation. Yao et al. have developed a polydopamine coating containing an antimicrobial peptide (AMP) for prolonged resistance to infection and prevention of crusting (Fig. 28) [138]. Cu^2+^-coordinated dopamine self-polymerization was used to improve the stability of the polydopamine coating on polymeric stents. AMP terminated with cysteine was further improved by the introduction of AMP on the surface of polydopamine. The Cu-coordinated polydopamine AMP coating on stents displayed good biocompatibility and could significantly suppress bacterial growth (*E. coli* and *P. mirabilis*) and finally reduce the deposition of struvite, biofilm formation, and hydroxyapatite crystals both *in vitro* and *in vivo*.

## 6 Otolaryngology stents

Stent implantation includes sinus stents, eustachian tube stents, endonasal stents, and salivary duct stents, which are advanced therapeutic strategies that offer unique advantages in otorhinolaryngology. Implantable drug delivery is an effective way to enhance these therapeutic features. Sinus stents have been proven effective in protecting against sinus outflow tract obstruction following functional endoscopic sinus surgery. Keeping the frontal sinus outflow tract open is uniquely challenging, and stenting and drug delivery are likely to be beneficial in this regard [139]. Drug-eluting stents are a promising option for the treatment of recurrent nasal polyposis as they increase systemic absorption compared to topical nasal steroid sprays and rinses.

**Fig. 29:**
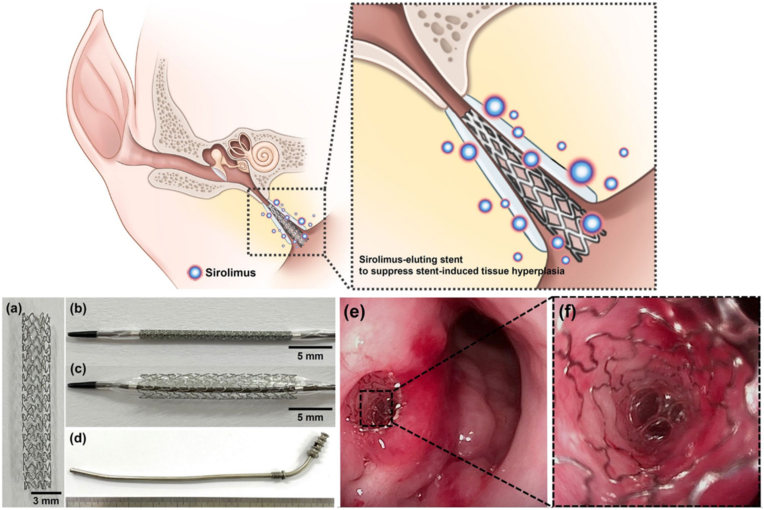
An eustachian tube stent made from a cobalt-chromium alloy with sirolimus eluting properties dysfunction treatment [140].

Currently, the eustachian tube (ET) stenting has been applied in ET dysfunction treatments. Unfortunately, these treatments are limited by ET obstruction and stent-induced tissue hyperplasia, infection and permanent perforation of the tympanic membrane. A sirolimus eluting cobalt-chromium alloy stent to suppress tissue and neointimal hyperplasia-induced in-stent restenosis was developed by Kang et al. (Fig. 29) [140]. In a pig model of the eustachian tube, stenting was successful in all ETs without complication and effectively inhibited submucosal fibrosis for 4 weeks of indwelling stent duration. However, mucus accumulation in and around the stent was observed with bare stents and the cobalt-chromium alloy-coated stent. Consequently, further studies are needed to verify antiproliferative drugs and the best stent materials.

## 7 Neural scaffold and lymphatic stents

**Fig. 30:**
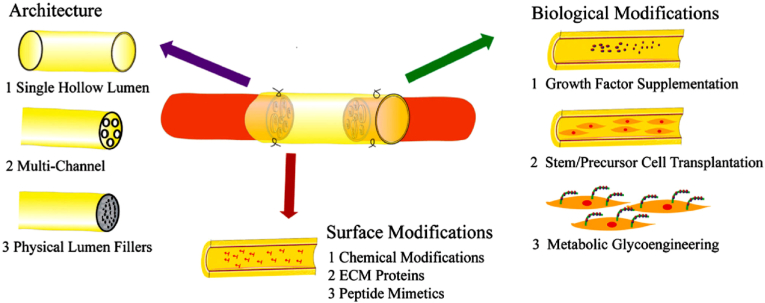
Schematic diagram of surface modification methods for various neural scaffolds [141].

Trauma-associated peripheral nerve defect is a widespread clinical problem. Emerging studies showed that neural scaffold is a feasible alternative to autologous nerve grafting for bridging nerve gap, establishing regenerative tunnel and improving functional recovery. An ideal neural scaffold should obtain optimal material, size, architecture, mechanical properties and surface physicochemical characteristics to be fully efficacious in nerve regeneration therapy. Obviously, previous studies have demonstrated that the specific configuration (e.g. hollow luminal NGC, Filaments-containing NGC, sponge-containing NGC, multichannel NGC), surface features (e.g. surface topography, charge, roughness) and microenvironment signals (e.g. chemical modification, grow factors, ECM proteins, petide mimetics) (Fig. 30) [141] of scaffold materials can effectively mediate adhesion, spreading, migration, morphology and specific gene expression of neural stem cell [142]. Certainly, there are still other prospective cues in state-of-art neural scaffolds that can be integrated with lumen surface modifications and functionalized coating containing newly developed drug and growth factors delivery systems.

Thoracic duct (TD) stenting is considered a promising technique for many lymphatic abnormalities and certain pathological conditions caused by TD obstruction and disruption, such as chyluria, plastic bronchitis and high lymph pressure [143]. However, bare stent occlusion in the TD may be caused by fatty debris or thrombus within the venous segment [144]. The surface modification and structure optimization of bare stent are critical issues for controlling symptoms recurred and retaining long-term patency. The treatments of stent occlusion has been described in a few reports so far.

## 8 Summary and perspectives

Stent implantation is beneficial for resolving the local obstruction and restenosis therapy. In clinical practice, the primary types of stents include cardiovascular stents, digestive tract stents, respiratory stents, reproductive system stents, and otolaryngology stents. The goal of stent implantation is to predominately maintain outstanding biofunctionality and desirable material-tissue interactions over extended periods. In addition, complications associated with bare stents are issues that must be addressed. Regarding the development of restenosis-resistant stents, there may be two directions in the future: structural design and surface functionalization of scaffold skeletons. To avoid the aggressive use of systemic drugs to prevent complications, biofunctional coated stents and drug-eluting stents are commonly used to treat local lesions.

In general, restenosis due to thrombosis and neointimal hyperplasia are the main causes of angioplasty stent failure. Stents that function in contact with blood generally lead to thrombosis. The drug eluting stents are still at a hotstage with a dramatic increase in the number of extensive scientific research. The development of thromboresistant stents with antiplatelet agents, thrombolytic agents, and drug-loading coating release of anticoagulants has been ongoing over the past decades. A promising solution to minimize post-operative complications appears to be the local and sustained delivery of selective pleiotropic drugs to limit smooth muscle cell proliferation. Therefore, anti-inflammation, rapid re-endothelialization, and anti-thrombosis are the critical issues for the long-term efficacy of vascular stents. For stents, restenosis and thrombosis are the classic themes that run throughout the drug eluting stents research over the last 20 years, while the bioresorbable vascular stents, dual antiplatelet therapy, antiplatelet drug and biodegradable polymer have the potential to become active topics and hotspots for future research. Blood-stent interactions and mechanisms of in-stent restenosis should be further progressed for guiding the microstructure design and manufacturing technology of bionic vascular stent design, multifunctional drug coatings on stents and more scientific postoperative interventions.

Over time, tumor overgrowth or inward growth can cause bare digestive tract stents to become blocked, tissue proliferation, and cholestasis, leading to progressive obstruction and failure. Recently, the most effective treatment seems to be using drug-coated stents for local drug delivery and anticancer therapy, which provides immediate relief of stenosis symptom and further applied chemotherapeutic action at the site of the tumor to inhibit the growth of the tumor with minimal side effects. Moreover, when the stent is removed after treatment, it is often embedded in surrounding tissue, making the intimal walls highly susceptible to rupture because it is difficult to separate the stent from the tissue. Pain/discomfort and secondary damage are usually seen after stent removal. To address these issues, new types of absorbable and biodegradable drug-loading stents would facilitate stent removal. Based on the development of the clinical needs and the research techniques of pancreatic/biliary stents, several possible directions for future development might include anti-protein coatings, anti-biofilm coatings, anti-biliary adhesion coatings, anti-tumor coatings, multifunctional coatings, and drug-eluting biodegradable stents.

In digestive tract therapy, stent-induced inflammatory reaction, granulation tissue hyperplasia, and sputum stasis have significantly affected the airway restenosis. Therefore, biodegradable materials and surface modification processing can reduce the possibilities of stent restenosis and complications. The occurrence of urothelial hyperplasia and bacterial contamination are common adverse effects and complications of ureteral stenting. There is a high likelihood of incrustation, which can become obstructive. Functionalized stents can reduce stent infection and stent encrustation. Some drug-eluting stents can sustainably deliver drugs to local lesions to suppress ureteral strictures and tumor growth. Furthermore, in otolaryngology treatments, common sinus stent insertion is limited by stent-induced tissue hyperplasia, infection and obstruction. There are unique challenges to maintaining the patency of the sinus outflow tract, and the antiproliferative and antimicrobial drug-eluting stents may be competitive solutions to tackle these challenges.

Despite the critical milestones in the development of cardiovascular stent and digestive tract stents, stent implantation-associated complications remain problematic. Stenosis of the respiratory stents, reproductive system stents, and otolaryngology stents is a substantial threat to human health and life. These stents are currently under development and yet show relatively slow progress. The interaction between the stent surface and the surrounding tissue must be taken into account in order to meet the long-term functional requirements with low complications. Surface-functionalization is a potentially promising measure for the regulation of interface behaviors. It is our expectation that this review would assist us in the development of effective, safe, and stable surface-engineered stents for the long-term treatment of occlusions.

## CRediT authorship contribution statement

**Yanghui Wen:** Writing – review & editing, Funding acquisition. **Yihuan Li:** Writing – review & editing, Data curation. **Rui Yang:** Writing – review & editing. **Yunjie Chen:** Funding acquisition. **Yan Shen:** Investigation. **Yi Liu:** Project administration, Funding acquisition, Conceptualization. **Xiaomei Liu:** Data curation. **Botao Zhang:** Formal analysis. **Hua Li:** Funding acquisition.

## Declaration of competing interest

The authors declare that they have no known competing financial interests or personal relationships that could have appeared to influence the work reported in this paper.

## Data Availability

Data will be made available on request.
